# Methodological Considerations in Conducting an Olfactory fMRI Study

**DOI:** 10.3233/BEN-120320

**Published:** 2013

**Authors:** Faezeh Vedaei, Mohammad Fakhri, Mohammad Hossein Harirchian, Kavous Firouznia, Yones Lotfi, Mohammad Ali Oghabian

**Affiliations:** ^1^Neuroimaging and Analysis Group (NIAG), Research Center for Molecular and Cellular Imaging (RCMCI), Tehran University of Medical Sciences, TehranIran; ^2^Advanced Diagnostic and Interventional Radiology Research Center, Tehran University of Medical Science, TehranIran; ^3^Iranian Center of Neurological Researches, Tehran University of Medical Science, TehranIran; ^4^University of Social Welfare And Rehabilitation Sciences, TehranIran

**Keywords:** Olfactory system, neuroimaging, task design, fMRI

## Abstract

The sense of smell is a complex chemosensory processing in human and animals that allows them to connect with the environment as one of their chief sensory systems. In the field of functional brain imaging, many studies have focused on locating brain regions that are involved during olfactory processing. Despite wealth of literature about brain network in different olfactory tasks, there is a paucity of data regarding task design. Moreover, considering importance of olfactory tasks for patients with variety of neurological diseases, special contemplations should be addressed for patients. In this article, we review current olfaction tasks for behavioral studies and functional neuroimaging assessments, as well as technical principles regarding utilization of these tasks in functional magnetic resonance imaging studies.

## 1. Introduction

In the last two decades, many studies with diverse methodologies have been carried out to establish neuronal correlates of the olfactory perception in the human brain. Thus far, cerebral brain imaging techniques especially functional magnetic resonance imaging (fMRI) and positron emission tomography (PET) have been used to investigate the regions involved in sensory processing such as olfaction [1,2]. Although few brilliant studies have focused on functional imaging of brain activation by odorants in humans [3,4], we believe that functional imaging of the olfactory system still remains understudied. Here, we put emphasis on the methodological approaches that should be considered in these studies. To achieve this goal, we briefly review olfactory processing and activation maps that yielded in the current olfactory fMRI literature. Then, methodological considerations for conducting an fMRI study for evaluation of the olfactory system will be proposed. Throughout this review, a contemplative look is used to highlight differences between studies on healthy and patient population.

## 2. Olfactory system

The olfactory system is a chemosensory apparatus that processes a wide range of information with regard to the identity, concentration and quality of the chemical stimuli. Axons leaving the olfactory epithelium accumulate into collections that exit the ciribriform plate of the ethmoid bone to reach the olfactory bulbs. The projections to the limbic system components have widespread interconnection with many parts of the brain. Of these parts mediodorsal thala-

ISSN 0953-4180/13/$27.50 © 2013 – IOS Press and the authors. All rights reserved mus, hypothalamus, frontal and temporal cortices are of great importance [2,5].

During smelling, brain is involved in perception, discrimination, and recognition of the odor [3]. Hence, compared to the studies that are focused on other sensory systems, when conducting functional or behavioral studies that focus on the olfactory system, defining a specific question/aspect of the olfactory system is more important.

## 3. Brain mapping using fMRI

Compared to other functional imaging techniques, Magnetic Resonance Imaging (MRI) has the advantage of pairing T1-weighted anatomical imaging with T*2-weighted functional imaging. This advantage makes it possible to generate simultaneous functional and anatomical maps for a certain subject. Functional magnetic resonance imaging (fMRI) is based on paramagnetic properties of oxygenated and deoxygenated hemoglobin that expresses changes of blood flow in response to coherent neural activity [6].

FMRI typically uses blood oxygen level-dependent (BOLD) contrast [6]. Alterations in regional blood volume or blood oxygenation following brain activation translate into changes in the BOLD signal, which signifies an indirect measure of neuronal activity. This relationship has resulted in tremendous research on neurophysiological basis of the fMRI signal [7].

## 4. Odor perception in healthy subjects

During smelling of an odorant, various processing steps are done by human brain. The processes include perception, discrimination and recognition of the odor [3]. Core olfactory regions that are commonly recruited during smelling of an odorant include piriform cortex, amygdale, insula, orbitofrontal gyrus, cingulate cortex and right thalamus [8]. Apart of these shared regions that are involved during most of the olfactory tasks, a wide network of olfactory regions gets involved in olfactory processing that depends on the type of odorant and the task presentation [3]. This network seems to be organized in a parallel or hierarchical manner. Figure 1 shows the pathway of odor intensity discrimination (OD-i), odor quality discrimination (OD-q) and odor recognition memory (OM) [3,8].

It should be noted that sniffing and smelling are two separate functions involved during olfactory stimulation and employ separate parts of the olfactory cortex. Sniffing in a presence and absence of odorants induces activation in the piriform cortex and posterior orbitofrontal gyrus [9,10]. In principle, piriform cortex and other parts of the primary olfactory cortex are activated in a short time stimuli (less than 10–15 seconds), and then the BOLD signal decreases to baseline level. Habituation is the reason of reduction of neural activity in primary olfactory cortex [11]. Another reason that might be contributed to inconsistent activation of piriform cortex is the magnetic impressionability artifact, which may lead to losing the signals in these regions. In most of olfactory fMRI studies, activation of primary olfactory cortex seems slight or very inconsistent, because in these studies odorant stimuli presented for a long time [9–12].

As far as functional neuroimaging is concerned, characteristics of odorants such as their familiarity, pleasantness and subject's judgment about them are also important and these aspects might influence the activation maps in fMRI studies [13]. Plailly and colleagues [13] studied neural network of primary olfactory structures involved in familiarity judgment task. They found that right piriform cortex is activated during this task and this activation could be attributed to olfaction recognition memory. Thus, it is concluded that right hippocampus, left inferior frontal gyrus and mid-fusiform gyrus are participated in recognition memory [3,13–15]. In another study to highlight importance of stimulus characteristics in olfaction studies, Rolls and coworkers [16] have investigated brain regions involved in perception of pleasant and unpleasant odors. They showed significant activation in mediorostral of the orbitofrontal cortex by pleasant odors whereas no activity was seen in this region by unpleasant odors.

Although olfaction has a main role in perceiving and identification of odors, it has an additional function in action understanding and multimodal action integration. This aspect of olfactory system was highlighted in a study conducted by Tubaldi and colleagues [17]. That study revealed prominent activation in middle temporal gyrus and parietal cortex. These two areas are believed to play role as an integrative areas for multisensory cues such as visual, tactile, auditory and olfactory stimuli [17].

## 5. Olfaction processing in the presence of brain impairments

Investigations that used fMRI for understanding brain activations during olfactory tasks are not con-

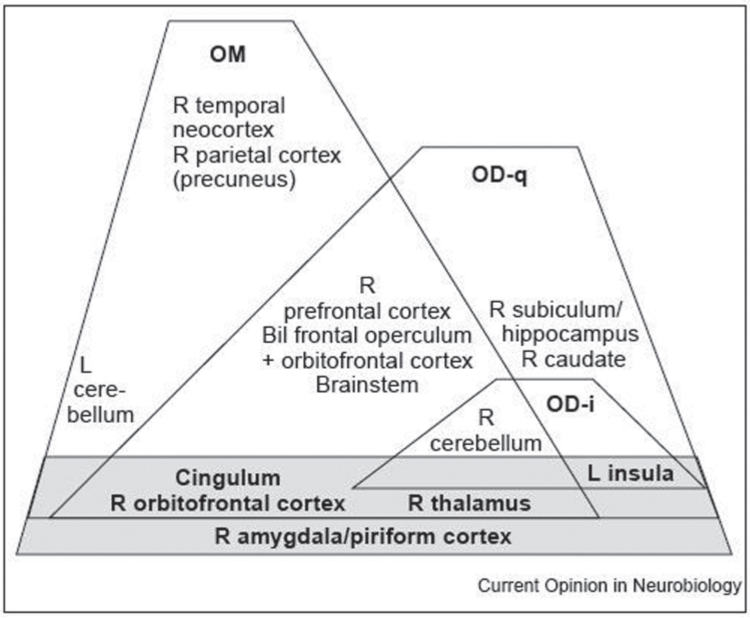

Fig. 1. A hierarchical and parallel system for odorant processing. This diagram depicts the activated regions shared by several olfactory tasks and the regions specific to a particular task. Each pyramid indicates a separate task. The higher up the pyramid, the more complex the task. The diagram reflects the parallel and hierarchical organization of the structures engaged, based on the anatomical connectivity data. The gray zone indicates the olfactory core regions, activated by passive smelling of odors. Bil, bilateral; L, left; R, right.

fined to normal subjects. Senile changes in the olfactory system, as well as olfactory function in certain diseases such as Schizophrenia, Parkinson's disease (AD) and Alzheimer's disease (AD), were also subjected to extensive research [18–20].

One of the factors that affect olfactory function is normal aging. According to a study conducted by Ducastel and colleagues [21], a significant reduction of activity in primary olfactory cortex (including piriform, amygdale, orbitofrontal cortex, insula and cerebellum) is evident in older subjects. Moreover, older subjects show a diverse activation map. The latter finding might be due to variability in compensation mechanisms for sensory deficits in those cases [21,22].

Schizophrenia is a disease that is showed to be associated with olfactory and emotional impairments. Schneider et al. [20] showed an olfactory dysfunction in these patients in regions involved in higher levels of olfactory processing including frontal, temporal and cingulated gyrus [20].

In a study conducted by Hummel and colleagues, olfactory function in patients with PD has been evaluated using fMRI. Brain activations with both pleasant and unpleasant odors were lower in amygdale and hippocampus in patients as compared to normal subjects. This finding could be justified by the diminished sensitivity of these regions to the emotional stimuli in the abovementioned disease [19].

Significant reduction in patient's ability to detect, recognize and discriminate between the odors in older subjects with mild cognitive impairments and patients with Alzheimer's disease have also been reported [18]. Olfactory dysfunction is a preclinical sign of cognitive diseases that arises at early stages of the disease progression [18,19,23,24]. Wang and colleagues [18] have recently compared AD patients and normal subjects and showed that brain activation in primary olfactory cortex (POC), insula, thalamus and hippocampus are lower in AD patients in comparison with the healthy group.

## 6. Technical Aspects

### *6.1. General considerations for fMRI studies*

There are numerous considerations in every fMRI study that are also important in fMRI studies of the olfactory system. Some technical considerations are related to the subjects. During an fMRI scanning, subjects should be asked to keep their head completely motionless and breathe normally and try to smell without sniffing [16,20,25,26]. Also, age is an important parameter that should be considered in fMRI tests. Subjects' ability to perform cognitive tasks may decline when they are older. This aspect is very important in designing olfactory tasks. Regarding a progressive reduction in subjects' ability to detect and identify odors, as they get older, it is necessary to choose suitable tasks that are compatible subjects' cognitive abilities [1,18,22].

### 6.2. *Stimulus delivery in olfactory experiments*

The most initial step in setting up an fMRI experiment for the olfactory system is to deliver odor molecules to the scanner room. One popular method is a piece of odor-saturated cotton that is presented in front of the subject's nose. This method is most appropriate in PET studies, whereas for fMRI, due to the length of the tunnel of the scanner, a plastic rod is preferred. This method is not very credible, because the odor concentration cannot be controlled. Moreover, the time of stimulus presentation is not precise and tactile stimuli may be happened if experimenter touches subject's nose. A more advanced method is to use dynamic olfactometer device, which is based on using odorless air pressure that carries the odorant molecules toward the subject's nose and hence it may overcome limitations of the previous method. Thus far, special olfactometers have been designed to generate olfactory stimuli in functional brain studies. These devices consist of a positive airway pressure device, a nasal mask and a unit that consists of odor-containing capsules. The system is completely controlled by a computer software that allows choosing specific switching between the odor and odorless phases, timing and frequency of olfaction stimuli [27,28]. Recently more convenient "mobile olfactory devices" have been developed. These stimulation devices are typically consist of three main parts including (i) a part for air entrance, control and distribution that is placed in the control room, (ii) odorants section, that is located in the magnet room and (iii) the delivery portion [29].

There are some technical considerations in olfactometer design that should be precisely regarded in fMRI studies. Those parts of the olfactometer device that are located in the magnet room should be made of diamagnetic matters to prevent any disturbance in the uniformity of the magnetic field and reduction of signal to noise ratio. Capability of delivery of various odors in a random fashion is another main characteristic of olfactometer device. A computer-based control allows the olfactometers to provide odor delivery in selectable durations [30]. Odor concentration is also important when olfactometer is to be used. The concentration of odors should be set to a known value. Moreover, since in most of the olfactory tasks there exists a "rest" or odorless state, this phase should only contain pure air that is not polluted by previously presented odor molecules. To avoid thermal and tactile stimulation during scanning, both odor and pure air that are conveyed to subject's nose must be at the same temperature and pressure [28,31].

Given the presence of body odor during the fMRI experiments and different processing of body odor as compared to common odors [32], special attention must be paid to proper delivery of the target stimulus during the scanning session. Indeed, if stimulus is not strong enough to overcome the body odor, the resultant activation map might be confounded by brain regions that are not related to the utilized task. This difference might be attributed to a central feature of odor perception, which is its hedonic or affective component [33]. Thus far, it has been stated that body others might deactivate (baseline versus body odors) anterior parts of the orbitofrontal cortex [32]. Hence, dominancy of the desired stimulus during olfactory studies along with careful interpretation of the activations maps is of great importance.

### 6.3. *Properties of the stimuli and rout of administration*

Stimulus to activate the olfactory-related regions in the brain could be delivered via three distinct structures including olfactory, trigeminal and vomeronasal systems [25]. Some odors are unimodal and they only activate the olfactory system. This is while most odorants are bimodal; they activate not only the olfactory system, but also the trigeminal nerve by free nerve endings and receptors located between the olfactory and respiratory epithelia [25,34].

When a desired odor is to be presented to a subject, stimulation could be achieved either by means of olfactometer device or through oral stimulation. In some studies subject's mouth is the primary rout of odor presentation. For example, Ducastel and colleagues [21] used a dissolved odorant in distilled water and then presented it to the subject's mouth by means of plastic tubes. Subjects were placed at the end of the tubes. Then, boluses of 50 $\mu$L of the odorant-containing solution were delivered every 3 seconds through automated syringes. So that, water and odor stimuli alternatively delivered to the tip of the tongue. This was occurred before the liquids being swallowed [21,35].

### 6.4. Considerations in image acquisition

One of the most important features in utilizing fMRI tasks is the synchronization between acquisition of the MR signal and stimuli transference [36]. Synchronization is especially important in complex designs to avoid loss of information. This issue has been addressed in various studies. Borromeo has shown that there is a lag between administration and presentation of odor in fMRI studies. Also a delay time of 12 seconds after the deactivation of stimuli has been reported in this study [36]. In general, activation of primary olfactory cortex could be missed due to habituation of these areas and lack of synchronization between presentation and administration of odor [11]. Therefore, in event-related and short time stimulation studies, it is important to eliminate the confounding effects of suspension time between odor presentation and odor delivery to the subject. To overcome this limitation, the air pressure should be controlled by adjusting the flow rate up to 7 L/min. Also, utilization of appropriate oil odorants with adjusted concentration can obtain a delay time less than 3 seconds. This time is more longer for liquid odorants [36].

### 6.5. Behavioral assessment

A behavioral assessment before functional brain imaging of the olfactory system helps to recognize olfactory abilities of the participants. Understanding the olfactory status of the subjects will help investigators for a better interpretation of the results in functional brain studies. On the other hand, it is useful to improve the quality of medical diagnosis and quality control of treatment of smell disorders [37]. Multiple standardized tests for assessment of olfactory function have been introduced. Here, we review more common validated tests that have been performed in most functional olfactory studies. Most of the tasks used in the current neuroimaging studies of the olfactory system root in their behavioral counterparts [37]. Hence, a rapid review of these traditional psychophysical tests of the olfactory system helps designing a better functional neuroimaging task.

#### 6.5.1. Sniffin' Stick

"Sniffin' Stick" test is introduced by Hummel in 1997 and is one of the most informative tests for estimating olfactory abilities [37]. "Sniffin' Stick" is a pen-like odor-dispensing device. This test is composed of three investigations of olfactory function including odor threshold (T), odor discrimination (D) and odor identification (I). In all of these tasks subjects should be blindfolded to prevent visual detection of target sticks. Using the three scores, a total score is determined by sum of the threshold, discrimination and identification test scores (TDI score). Accordingly olfactory function could be classified as anosmia (TDI $<$ 16), hyposmia (16 $<$ TDI $<$ 30) and normosmia (TDI $>$ 30) [19,23,37–43].

#### 6.5.2. University of Pennsylvania Smell Identification Test

The so-called UPSIT (University of Pennsylvania Smell Identification Test; Doty, 1989) is a 'scratch and sniff' test introduced in North America for odor identification [44]. This test is established upon microencapsulated odors that are released from the surface of strips via a pencil. Then, subject characterizes 40 odorants from a list of multiple-choice. The UPSIT is sensitive to gender, age, smoking or other habits and a number of olfactory disorders [18,37,45–47].

The Brief Smell Identification Test (B-SIT) is a shortened version of UPSIT [48]. The B-SIT includes a package of 12 odorants implanted in scent strips and are liberated by scratching with a pencil tip. The B-SIT score is defined as number of correctly identified answers. An impaired olfactory function is defined as a score below 9 [48].

#### 6.5.3. The San Diego Odor Identification Test

San Diego odor identification test is assessed using eight common natural odorants (e.g. coffee, chocolate, peanut etc.). In this test, the time interval between presentation of each odorant should be set to 45 seconds. After presenting all odorants in a randomized manner, participants could be presented with the misidentified odorants again to let them learn the unfamiliar odorants. The SDOIT score is obtained from sum of number of odorants (0 $<$ SDOIT score $<$ 8). Olfactory impairment is defined if correct identification would be less than 6 odorants [21,48,49].

### 6.6. Task design in olfactory studies

Using the abovementioned behavioral tests, many studied have designed fMRI tasks to activate brain networks during olfactory functions. Two different models can be used in fMRI studies to design stimulation paradigm. "Block designs" and "event-related" models are the two normative approaches to present the stimulus [36]. These two methods are also commonly used to activate neural networks of olfactory system during functional brain imaging. Block design, as a common method, is consisted of several activation blocks lasting 10 to 30 seconds [50]. After each activation block, a rest block is alternatively introduced to the subject. This interval should be longer than activation time to let BOLD signal to be decreased to its baseline level. Hence the rest block can be set to 30 to 60 seconds. It has been shown that a prolonged olfaction stimulation during fMRI imaging (for example 40 seconds of odor presentation and 40 seconds of a rest period) motivates a sharp increase of BOLD signal in POC that pursues a decrease to baseline level within 30–40 seconds [51]. Sobel et al. showed that this decaying response signal could be modeled by an exponentially function that possesses strong activation in POC (olfactory tubercle, piriform cortex, enthorhinal cortex, insula and amygdala) [50]. Hence, habituation to the stimulus is an important issue in block designs. Poellinger and colleagues [11] investigated effects of habituation in an odorant-induced stimulus within 60 seconds, while the odorless phases were set to 120 seconds. They used several analysis paradigms including 120; 60; 15; and 9 seconds to filter the time series signal in ON (activation) period to elicit activation patterns that were induced by the odorant. According to their experiment, the time course of the BOLD signal in piriform cortex is habituated very quickly and its activation lasts for 15 seconds. Afterwards it is decreased to the baseline level. Also they have concluded that only a 9-second ON paradigm could model early habituation in POC. In another study, Tabert and colleagues showed that the best model to fit habituating time course of the POC is a block of a 6-second stimulus presentation [11,52]. Tabert and his coworkers also revealed that the optimal model to fit the odor-induced activation in POC is a 6-second stimuli, while the 12 seconds model detected the odor-induced activation in higher olfactory regions (i.e. cingulated cortex, insula and orbitofrontal cortex) [52].

Other fMRI studies have shown the effective treatment of event-related designs in visualizing olfactory-related activations and the role of these designs to remove confounding effects of habituation in primary olfactory areas. However, block designs with prolonged stimulation are still routinely used in clinical studies [50,52–56].

Since the length of fMRI experiment is a limiting factor in task design, to achieve reliable and accurate results, the number of repetitions of odor stimuli should be optimized. Bitter and colleagues [57] have shown that the optimum olfaction stimuli repetitions to reduce olfaction habituation and provide high quality results should not be set to more than 4–8 times [57].

Application of odors in olfactory studies depends on the purpose of the study. Here we review some studies that focused on stimuli properties as the primary question in their task design. For example some reports have highlighted effects of hedonic valence and trigeminal component of odorants in brain network engagement. Rolls and colleagues [16] have investigated brain activations in response to pleasant and unpleasant odors in a normal group. They chose three pleasant odorants including alpha-ionone (food-related, woody), linalyl acetate (floral, sweet) and geranyl acetate (floral) and unpleasant odorants including isovalertic acid, hexanoic acid and octanol. Propylene glycol was used to dilute all odorants. Stimulus pulses had duration of 8 seconds for any odorant followed by a 24-second odorless interval. All odorants presented 10 times in a random block design [16]. In another study, Lombion and coworkers [25] studied brain activations in healthy subjects in response to odorants that activate both olfactory and trigeminal systems. Phenyl ethyl alcohol (rose-like odor) and isoamyl-acetate (banana-like odor) were chosen as a pure olfactory stimulus and a bimodal olfactory-trigeminal stimulus, respectively. Each participant encountered a 7-minute and 12 seconds fMRI scan compromised of 12 odorless epochs (27 seconds for each epoch) and 12 stimulus epochs (9 second for each epoch) by two odorants alternatively with odorless epochs [25]. It should be noted that the concentration of odor is also important. Quality of perception of odors is highly dependent on the odor concentration. For example, at low concentrations, indole smells as a floral odor whereas at higher concentrations it smells rotten [2].

### 6.7. *Olfactory tasks for patient population*

The abovementioned methodological considerations in task design for olfactory studies should be also considered in studies on patient population. In certain cognitive and psychological diseases, the olfaction's neural network will be impaired as a consequence of the disease course. Hence, reviewing tasks that were used in previous studies on patient population may highlight slight differences between task designs for healthy subjects and those with brain impairments.

Wang and colleagues [18] have investigated brain activations in response to three levels of concentration of lavender oil in the Alzheimer's disease patients and healthy subjects. Different concentrations in that study were 0.1%, 0.32% and 1.0% of lavender oil. The odors were presented in a 6-second block design and the resting state period (odorless air) was lasted for 42 seconds. Each concentration presented for three times and the trial was started with the lowest concentration. In that study the researchers have found that there was a significant correlation between BOLD signal intensity and the odor concentration in AD patients within the POC. On the other hand, no difference in BOLD signal intensity was observed with different odor concentrations in the healthy group [18].

As it is discussed earlier in this paper, in PD, a kind of anosmia is usually encountered at early stages of the disease. Hummel et al. [19] have investigated brain activity in response to pleasant (phenyl ethyl alcohol) and unpleasant (hydrogen sulfide) odors in PD patients. For each condition participants were examined by a block of 1-second stimulation and a 2-second interval between the stimulations that were repeated for 10 times. This activation period was followed by a 32-second rest period (with pure odorless air). Each session consisted of six stimulation periods that were lasted for approximately 6 minutes [19,38].

Usage of olfactory tasks in fMRI is not confined to Alzheimer's and Parkinson's diseases. Schneider and colleagues in [20] investigated neural substrates of the odor processing in Schizophrenia. In that study, for each participant, brain activation was assessed in response to positive (vanilla), negative (rotten yeast) and neutral (ambient air) olfactory stimuli. FMRI was administered in a block design that was comprised of three conditions (positive, negative and ambient air) so that the neutral stimulation presented between two other conditions. Each condition consisted of three odorant stimulus phases presented only within the first 2 seconds of each repetition time (TR = 4 seconds). The four rest period phases were lasted for 40 seconds. In their suggested task, each condition took approximately 4 minutes and 52 seconds [20].

According to this review, task design modeling is dependent on objectives of the study, subjects' characteristics and their probable cognitive impairments. One should ask which part of the olfactory system is more affected by a certain disease and the task design should focus on this question.

### 6.8. *Analysis approach*

After preprocessing steps, statistical analysis is performed to create activation maps and determine active voxels in response to the stimulus. Applying special thresholds on the statistical maps reveals only those voxels with greater activation than the specified threshold. The most common approach for data analysis in functional brain imaging is a model-based statistical analysis called "general linear model" (GLM) [58,59]. GLM applies and fits a linear model to the data to estimate the expected pattern of activation [59].

To achieve a model that best fits to a data, stimulus paradigm function should be convolved with the hemodynamic response function (HRF). The importance of this achievement is to justify the time course of the stimulation paradigm and the neural response of the brain [52,59]. In principle, model-based methods extract brain responses by correlating signal changes of each voxel with the time course of the task template convolved with the hemodynamic function. Olfaction perception is more time consuming than visual and auditory perceptions. This could be attributed to the adaptation and habituation of the odor perceiving. Previous studies have proposed an optimal template to extract a brain signal in response to olfactory stimulation. Ducastel and colleagues [35] have compared activation patterns obtained from perception-based paradigms in response to odor stimulus (ethyl butyrate) and the template-based models. According to the findings of that study, stronger activation can be observed by the perception-based templates than with the stimulation-based templates. Perception-based templates had shown significant activations in the left piriform cortex, left inferior frontal gyrus, and the right mesiotemporal lobe. These activations were not encountered in the stimulation-based templates [35].

In many studies, region of interest (ROI) analysis is used to make a more accurate understanding of the activated regions in statistical maps. Moreover, in complex designs with various conditions, it is difficult to extract the activation pattern from an overall map. For such cases, ROI analysis could be used to see the signal for each condition across the region of interest. Also this method reduces unnecessary analyses and statistical tests could be confined to certain regions [60].

In olfactory fMRI studies, several regions might be used to mask the activation maps and determine the degree of correlation between analyses in different conditions [21]. Main regions of interest in olfactory studies include amygdale, enthorhinal cortex, piriform cortex, hippocampus, parahippocampus, uncus, insula, orbitofrontal cortex, anterior and posterior cingulated cortices. These anatomic areas are usually defined according to Thalairach atlas and its coherent coordinates [21,22,35]. The aforementioned areas were obtained from several fMRI studies that have been conducted to define most important regions with regard to olfactory processing. So far, Poellinger and colleagues showed that piriform, entorhinal cortex, orbitofrontal cortex, hippocampus, insula, medial dorsal thalamic, cingulated cortex, frontal operculum, caudate nucleus, putamen and superior temporal sulcus are among most important ROIs in a habituation olfactory task [11].

Validity of ROIs is directly related to the studies' population and could be different for certain diseases. As an example, Schneider et al. investigated brain activations in response to odor stimulation in Schizophrenia patients and compared the results to those of healthy volunteers. That study concluded that 9 different volumes of interest (VOI) could be explored in schizophrenic patients. These areas were defined on the basis of pathophysiology of schizophrenia. The regions involved in olfaction and emotional processing included thalamus, anterior cingulated gyrus, orbitofrontal gyrus, middle frontal gyrus, temporal cortex, insula, amygdale, hippocampus and nucleus accumbens [20].

Regarding a large diversity in fMRI studies of the olfactory system and their different experimental approaches, statistical analysis, and data interpretation, recently a meta-analysis have been conducted to provide a more general overview of structure and function of the primary and secondary olfactory networks [61]. Seubert et al. used the Activation Likelihood Estimation (ALE) method to statistically merge all published functional olfactory neuroimaging studies and report a comprehensive probability map. Using "GingerALE transformation tool" and applying odor versus baseline contrast, this meta-analysis has shown the olfactory bulb, piriform cortex, insula, amygdale, orbitofrontal cortex and ventral putamen as primary and secondary olfactory cortices [61].

Another aspect in neuroimaging analysis is correlating activations maps with certain covariates. In most studies, linear regression is used to depict the correlation between olfactory capabilities as an independent variable, and specific impairments as dependent variables. As an instance, Pollatos and colleagues [40] have found a significant correlation between olfactory sensitivity and depression symptoms. In that study, "Sniffin Stick" test was used to evaluate olfactory sensitivity in normal subjects. Meanwhile, "Beck Depression Inventory (BDI)" measured the depression scores in the patients. BDI scores were utilized to categorize depressive symptoms into mild, moderate and severe depressions. The correlation between depression symptoms and olfactory functions was examined with Pearson's correlation analysis and showed a negative correlation between olfactory sensitivity and depression symptoms [40,60]. The use of disease scoring systems is not confined to the depressed patients. Wang and colleagues have shown that the BOLD signal activity in response to olfactory stimulus with low concentration of lavender (0.1%) is particularly correlated to UPSIT scores in the healthy and patients with Alzheimer's disease. Brain activations in response to low concentration of lavender (0.10%) was mainly observed in left hippocampus, left POC and left insula. This finding may imply that brain activity in these regions is significantly correlated with the olfaction identification abilities [18].

## 7. Conclusions

This overview exhibits how fMRI brain imaging can takes part in a better comprehension of functional brain in regard to olfactory stimulus. Since the introduction of functional brain imaging with MRI, several tasks were used to activate brain networks in both healthy subjects and patient population. Thus far there is a paucity of data with regard to use of olfactory stimulus to activate brain regions in the current literature. This may roots in technical difficulties in designing olfactory tasks. Due to the wealth of present data regarding impairments of olfaction in certain cognitive diseases, we reviewed commonly used tasks and methodological consideration in application of olfactory tasks in healthy and diseased subjects.

## Acknowledgment

This review was supported by the "Legal Medicine Organization, Islamic Republic of Iran".
